# Acute treadmill exercise discriminately improves the skeletal muscle insulin‐stimulated growth signaling responses in mice lacking REDD1

**DOI:** 10.14814/phy2.14011

**Published:** 2019-02-21

**Authors:** Cory M. Dungan, Bradley S. Gordon, David L. Williamson

**Affiliations:** ^1^ Department of Rehabilitation Sciences College of Health Sciences University of Kentucky Lexington Kentucky; ^2^ Department of Nutrition, Food, and Exercise Sciences College of Human Sciences Florida State University Tallahassee Florida; ^3^ Kinesiology Program School of Behavioral Sciences and Education Penn State Harrisburg Middletown Pennsylvania

**Keywords:** Aerobic exercise, FOXO, insulin signaling, mTORC1, REDD1

## Abstract

A loss of the regulated in development and DNA damage 1 (REDD1) hyperactivates mechanistic Target of Rapamycin Complex 1 (mTORC1) reducing insulin‐stimulated insulin signaling, which could provide insight into mechanisms of insulin resistance. Although aerobic exercise acutely inhibits mTORC1 signaling, improvements in insulin‐stimulated signaling are exhibited. The goal of this study was to determine if a single bout of treadmill exercise was sufficient to improve insulin signaling in mice lacking REDD1. REDD1 wildtype (WT) and REDD1 knockout (KO) mice were acutely exercised on a treadmill (30 min, 20 m/min, 5% grade). A within animal noninsulin‐to‐insulin‐stimulated percent change in skeletal muscle insulin‐stimulated kinases (IRS‐1, ERK1/2, Akt), growth signaling activation (4E‐BP1, S6K1), and markers of growth repression (REDD1, AMPK, FOXO1/3A) was examined, following no exercise control or an acute bout of exercise. Unlike REDD1 KO mice, REDD1 WT mice exhibited an increase (*P* < 0.05) in REDD1 following treadmill exercise. However, both REDD1 WT and KO mice exhibited an increase (*P* < 0.05) AMPK phosphorylation, and a subsequent reduction (*P* < 0.05) in mTORC1 signaling after the exercise bout versus nonexercising WT or KO mice. Exercise increased (*P* < 0.05) the noninsulin‐to‐insulin‐stimulated percent change phosphorylation of mTORC1, ERK1/2, IRS‐1, and Akt on S473 in REDD1 KO mice when compared to nonexercised KO mice. However, there was no change in the noninsulin‐to‐insulin‐stimulated percent change activation of Akt on T308 and FOXO1/3A in the KO when compared to WT or KO mouse muscle after exercise. Our data show that a bout of treadmill exercise discriminately improves insulin‐stimulated signaling in the absence of REDD1.

## Introduction

Approximately 25 million Americans are diagnosed with diabetes (American Diabetes A, 2018) which impacts the normal physiologic response to insulin‐stimulated glucose uptake (Hansen et al. 1995; Wang et al. 1999), lipid storage (Porstmann et al. 2008), protein synthesis (Vander Haar et al. 2007), and cell growth and survival (Brunet et al. 1999) among other functions. Insulin exerts its cellular function by binding to the insulin receptor and activating a downstream cascade through insulin receptor substrate‐1 (IRS‐1) (Sun et al. 1991). IRS‐1 can stimulate downstream signaling kinases, including protein kinase B/Akt (Vanhaesebroeck and Alessi 2000) and the mitogen‐activated protein kinase (MAPK) pathways to promote cell metabolism, growth, and proliferation (Weng et al. 2001). Akt activation indirectly promotes the activation of mechanistic target of rapamycin complex I (mTORC1) (Vander Haar et al. 2007) by phosphorylating the tuberous sclerosis complex 2 (TSC2) protein, inhibiting the TSC1/2 complex (Potter et al. 2002). Conversely, conditions that exhibit chronic hyperactivation of mTORC1 (e.g. obesity, aging, loss of TSC1) reduces IRS‐1 tyrosine activation during basal or fasted conditions, and limit the ability of insulin to activate IRS‐1 (Craparo et al. 1997). Akin to Akt, the stimulation by growth factors (e.g. insulin) multiple tyrosine sites on IRS‐1 are phosphorylated, initiates a phosphorylation cascade and the subsequent activation of the MAPK/Erk kinase (MEK1/2) and extracellular signal‐regulated kinases 1/2 (ERK1/2) proteins (Skolnik et al. 1993).

Aerobic exercise has been employed as a means to improve insulin sensitivity and glucose metabolism (Cartee et al. 1989; Ren et al. 1994; Kennedy et al. 1999) in specific populations at risk for reduced insulin sensitivity (i.e. obese, aged, diabetic). One of the mechanisms involved in reduced insulin signaling is mTORC1 hyperactivation, which inhibits insulin‐mediated IRS‐1 activation of inhibitory serine phosphorylation sites by the mTORC1 kinase, S6K1 (Aguirre et al. 2002; Tremblay et al. 2007). Aerobic exercise can inhibit mTORC1 activation by upregulating the stress activated protein regulated in development and DNA damage 1 (REDD1), a potent mTORC1 inhibitor (Hayasaka et al. 2014) that functions to limit growth during times of DNA damage, hypoxia, and cellular stress (Ellisen et al. 2002; Shoshani et al. 2002; Wang et al. 2003). REDD1 inhibits mTORC1 signaling by sequestering the 14‐3‐3 protein from tuberous sclerosis complex 1/2 (TSC1/2), another mTORC1 inhibitor, preventing Rheb‐GTP loading, and subsequent activation on mTORC1 (DeYoung et al. 2008). REDD1 also inhibits cellular growth and function by directly inhibiting Akt phosphorylation and activation via increased association of the phosphatase, PP2A, with Akt (Dennis et al. 2014). When REDD1 is ablated, mTORC1 is hyperactivated. Aberrant activation of mTORC1 such as this can significantly diminishing IRS‐1 tyrosine phosphorylation upon stimulation by insulin and other growth promoting stimuli (Regazzetti et al. 2012; Dungan et al. 2014). Part of improvement in insulin‐stimulated signaling with aerobic exercise, as it relates to mTOR, is also mediated by AMP‐activated protein kinase (AMPK). A high cellular AMP:ATP ratio brought on through ATP utilization by aerobic exercise activates AMPK (Ruderman et al. 2003; Wojtaszewski et al. 2003). AMPK activation can also inhibit mTORC1 by direct phosphorylation of the mTORC1 accessory protein, Raptor (Gwinn et al. 2008), and TSC1/2 (Inoki et al. 2003).

We previously reported that a lack of REDD1 reduces glucose tolerance and insulin‐stimulated signaling in skeletal muscle, which was associated with hyperactive mTORC1 signaling and potential negative feedback on IRS‐1 (Wilkinson et al. 2012; Dungan et al. 2014; Williamson et al. 2014). Moreover, our laboratory reported that acute rapamycin treated REDD1 knockout (KO) mice exhibited improved skeletal muscle signaling responses to insulin treatment (Dungan and Williamson 2017). Likewise, an acute bout of aerobic exercise effectively promotes insulin sensitivity (Ren et al. 1994; Kennedy et al. 1999) and REDD1 expression (Murakami et al. 2011; Hayasaka et al. 2014; Gordon et al. 2017) while reducing mTORC1 activation. However, the role of REDD1 on the aerobic exercise‐mediated responses to insulin in skeletal muscle has not been established. Therefore, the goal of this study was to determine if aerobic exercise is effective in improving skeletal muscle insulin‐stimulated signaling activation during a loss of REDD1. We hypothesized that an acute bout of treadmill exercise will reduce the basal hyperactive mTORC1‐associated signaling observed in skeletal muscle from REDD1 knockout mice, and subsequently improve insulin‐stimulated signaling responses when compared to nonexercised REDD1 KO mice.

## Methods

### Animals

The Institutional Animal Care and Use Committee at the University at Buffalo approved the protocols and procedures used in the studies herein. Subsequent analysis and manuscript preparation were performed at Penn State Harrisburg. All mice were housed at 21°C in 50% humidity with 12/12 h light/dark cycle on a standard chow diet (Harlan; Cat# 2018). 3–4‐month‐old REDD1 wild‐type and RTP801 (REDD1) knockout mice in a C57Bl/6x129SvEv background (generated by Lexicon Inc.; Woodland, TX for Quark Pharmaceuticals Inc.; Fremont, CA) (Brafman et al. 2004) that were initially provided by Drs. Elena Feinstein and Rubin Tuder, were used in this study (*N* = 5–6/group).

### Treadmill exercise

**Figure 1:**
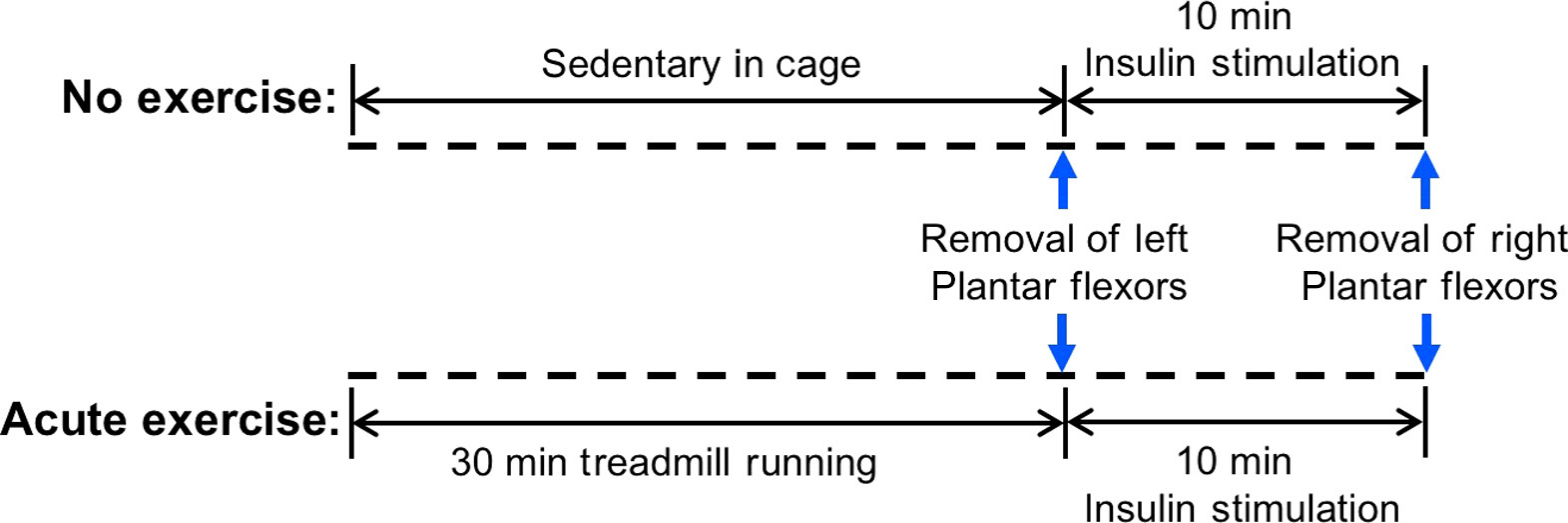
Schematic of the acute exercise and insulin injection protocol. Following a 3‐day acclimation period, groups of REDD1 wildtype (WT) and knockout (KO) mice were either exercised on a 5% grade at 20 m/min for 30 min or left sedentary in their cages. Immediately following the exercise bout, the left plantar flexors (soleus, plantaris, and gastrocnemius muscles) were removed. We then performed an intraperitoneal injection of insulin at a concentration of 0.5 IU/kg BW, waited 10 min, and then removed the right plantar flexor muscles.

Mice were exercised using a protocol that was adapted from one previously published by our laboratory (Williamson et al. 2006). Briefly, mice were acclimatized to the motorized treadmill (Columbus Instruments Exer3/6 Treadmill (Columbus, OH) by running 10 min/day for 3 days. The grade was held constant at 5%, while the speed was gradually increased to 20 m/min^−1^ over the 3 day period. The mice were fasted 3 h prior to the experiment. A 3 h fast was used because overnight fasting severely reduces mTORC1 activation (Williamson et al. 2014), as does aerobic exercise (Williamson et al. 2006). This was done to maintain the ability to observe changes in mTORC1 activation preexercise to‐postexercise, while partially normalizing food consumption. As depicted in Figure 1, the mice were run on a 5% grade at 20 m/min for 30 min.

### Insulin treatment

Immediately following the treadmill run, animals were anesthetized by 3.5% isoflurane anesthesia and the right plantar flexor complex (containing the medial and lateral gastrocnemius, soleus, and plantaris muscles) was removed and immediately placed it in liquid nitrogen. Insulin (Humulin, Eli Lilly) was then injected into the intraperitoneal space at a concentration of 0.5 IU/kg BW. After 10 min, the remaining (left) plantar flexor complex was removed and placed immediately into liquid nitrogen. Following the experiment, all samples were stored at −80°C for subsequent analysis (Dungan et al. 2014). We do acknowledge that the plantar flexor complex is a mixed muscle group. However, consistency with our previous reports (Dungan et al. 2014; Dungan and Williamson 2017) using this muscle group in these REDD1 WT and KO mice were paramount, since these findings served as the basis for this study. Also consistent with our previous reports (Williamson et al. 2014, 2016), the expression of noninsulin‐to‐insulin percent (%) change reported in the figures were insulin stimulated minus noninsulin stimulated divided by noninsulin stimulated times 100 for the respective kinase or protein.

### Tissue homogenization

The plantar flexor complex samples were homogenized in 10 volumes of CHAPS‐containing buffer [40 mmol/L HEPES (pH 7.5), 120 mmol/L NaCl, 1 mmol/L EDTA, 10 mmol/L pyrophosphate, 10 mmol/L glycerophosphate, 40 mmol/L NaF, 1.5 mmol/L sodium vanadate, 0.3% CHAPS, 0.1 mmol/L PMSF, 1 mmol/L benzamidine, 1 mmol/L DTT, and protease inhibitors (#04693116001, Roche, Indianapolis, IN)] (Dungan et al. 2014). The resulting homogenate was clarified by a 1000*g* centrifugation for 5 min (at 4°C), and the supernatant was retained (i.e. cytoplasmic fraction). A small aliquot of the cytosolic fraction was taken for the determination of protein concentration for each sample. Then, equal volume of 2X sodium dodecyl sulfate loading buffer was added and the samples underwent Western analysis or were stored at −80°C.

### Western blotting

30 μg of protein, as determined by a Coomassie/Bradford protein assay, was resolved using sodium dodecyl sulfate polyacrylamide gel electrophoresis (SDS‐PAGE), and then transferred onto PVDF membrane (Bio‐Rad Protean). After blocking in 5% milk in tris‐buffered saline (TBS) plus 0.1% Tween‐20 (TBS‐T) for 1 h at room temperature, membranes were incubated with primary antibody for overnight at 4°C in TBS‐T. Membranes were washed and incubated with a horseradish peroxidase (HRP)‐containing secondary antibody for 1 h in a 5% milk/TBS‐T solution at room temperature. Then membranes were washed in TBS‐T and prepared for imaging. Protein immunoblot images were visualized following the addition of Bio‐Rad Clarity ECL reagent and captured (Bio‐Rad ChemiDoc MP Imager). If required, blots were stripped and reprobed with antibodies that recognize the total form of a protein independent of phosphorylation state or GAPDH. Primary antibody for AMPK Thr172, 4E‐BP1 Ser37/46, S6K1 Thr389, rpS6 Ser240/244, MEK1/2 Ser217/221, ERK1/2 Thr202/Tyr204, IRS‐1 Tyr1222, Akt Thr308 and Ser473, FOXO1/3A Thr24/32, and GAPDH, were purchased from Cell Signaling Technology (Beverly, MA), and REDD1 was purchased from Protein Tech. Density measurements for the images were quantified using Bio‐Rad ImageLab software, and were normalized to the appropriate control. Each sample was then normalized to the WT group, for the respective blot, and then expressed as a mean percentage of the WT group between blots.

### Statistical analysis

**Figure 2:**
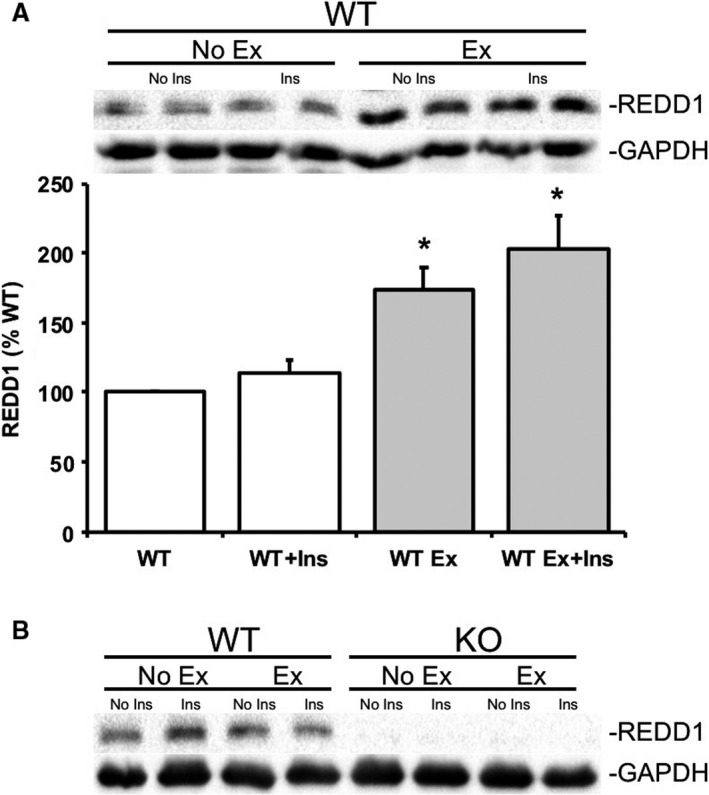
Acute treadmill exercise promotes REDD1 protein expression in REDD1 wildtype mice. Skeletal muscle from REDD1 wildtype (WT) mice were examined for (A) REDD1 protein expression and (B) REDD1 WT and REDD1 KO mice (KO) by Western blot analysis following either no exercise/no insulin (WT), after an acute insulin injection (WT + Ins), after an acute bout of treadmill exercise (WT Ex), or after an acute bout of treadmill exercise and insulin injection (WT Ex + Ins). Blots are normalized to GAPDH for each respective group. *P < 0.05 versus REDD1 WT group. Representative blots are shown. *N* = 5–6 per group.

Statistics were performed using IBM SPSS v24.0 software for Mac. A two‐way analysis of variance (ANOVA) was used to examine the differences between (WT, WT + EX, WT + Insulin, WT + EX + Insulin; Fig. 2 only) or within animal noninsulin‐to‐insulin‐stimulated percent change differences of protein expression/kinase activation for genotype (WT, KO) and exercise (no exercise, exercise). If significance was found, a Tukey's post‐hoc test was performed to determine significance between groups. The results are expressed as the mean ± SE with an *n* = 5–6 for each group. The significance level was set at *P* < 0.05.

## Results

**Figure 3:**
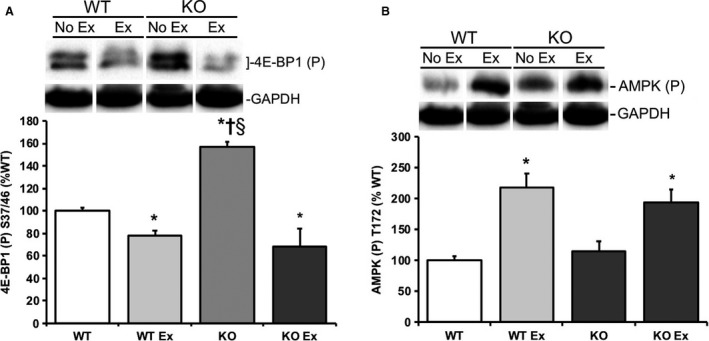
REDD1 KO mouse muscle exhibited aberrant basal/fasted 4E‐BP1 phosphorylation through appropriate AMPK phosphorylation following acute treadmill exercise. Skeletal muscle were examined for (A) phospho 4E‐BP1 S37/46 and (B) phospho AMPK T172 following either no exercise/no insulin in REDD1 WT mice no exercise/no insulin (WT), after an acute exercise bout in REDD1 WT mice (WT Ex), no exercise/no insulin in REDD1 KO mice (KO), after an exercise bout in REDD1 KO mice (KO Ex) by Western blot analysis. Blots are normalized to GAPDH for each respective group. *P < 0.05 versus REDD1 WT group, ^†^ *P* < 0.05 versus REDD1 WT Ex group, ^§^ *P* < 0.05 versus REDD1 KO Ex group. Representative blots are shown. *N* = 5–6 per group.

Initially we confirmed previous reports (Murakami et al. 2011; Hayasaka et al. 2014; Gordon et al. 2017) that an acute bout of treadmill exercise promoted a significant elevation (*P* < 0.05) of REDD1 protein expression in REDD1 WT mice (Fig. 2A), which was independent of acute insulin treatment (Fig. 2A). Consistent with our previous work (Dungan et al. 2014; Williamson et al. 2014; Dungan and Williamson 2017), REDD1 protein expression was not detectable in REDD1 KO mouse skeletal muscle during either a basal/fasted state, after an acute bout of treadmill exercise, or after insulin treatment when compared to REDD1 WT mouse muscle (Fig. 2B). REDD1 KO mouse muscle also exhibited a significant basal/fasted hyperactivation (*P* < 0.05) of the mTORC1 signaling kinase, 4E‐BP1, under a state when compared with basal/fasted REDD1 WT mice (Fig. 3A). Though following a bout of treadmill exercise, 4E‐BP1 phosphorylation was significant lower (*P* < 0.05) in REDD1 WT and KO muscle when compared to REDD1 WT or KO, nonexercised mice (Fig. 3A). Conversely, skeletal muscle AMPK T172 phosphorylation was significantly higher (*P* < 0.05) in both REDD1 WT and KO mouse muscle (Fig. 3B) immediately following an acute bout of treadmill exercise when compared to the respective genotype, nonexercised mice.

**Figure 4:**
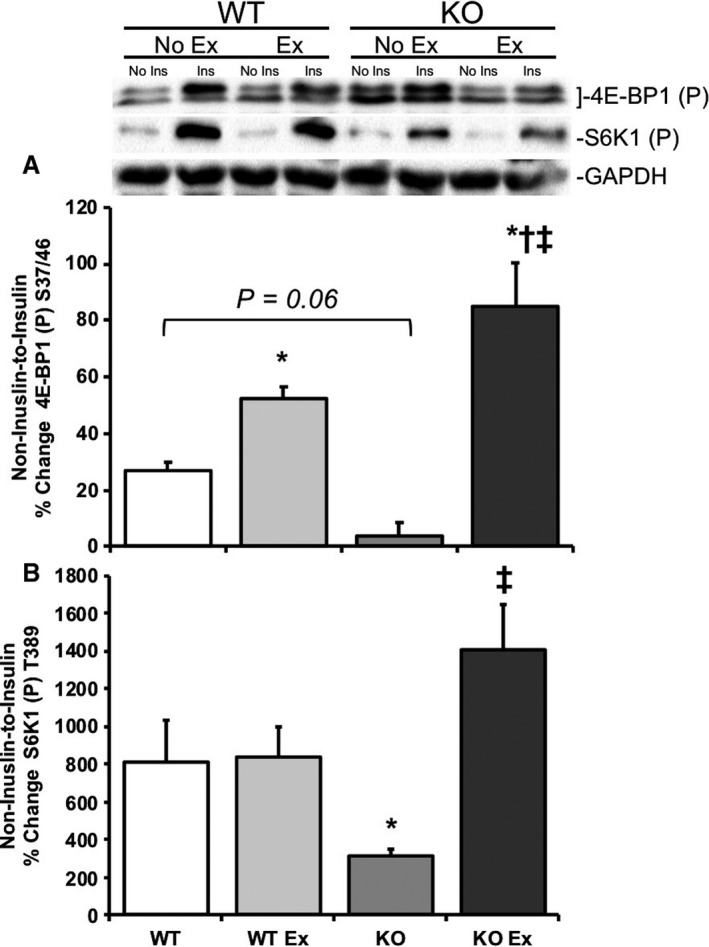
Acute treadmill exercise improves the noninsulin‐to‐insulin‐stimulated mTORC1 signaling in REDD1 KO mouse skeletal muscle. Skeletal muscle from REDD1 WT mice after no exercise −/+acute insulin injection (WT), after an acute exercise bout −/+acute insulin injection in REDD1 WT mice (WT Ex), REDD1 KO mice after no exercise −/+acute insulin injection (KO), and REDD1 KO mice after acute treadmill exercise −/+acute insulin injection (KO Ex) were examined for (A) the noninsulin‐to‐insulin‐stimulated percent change for phospho 4E‐BP1 S37/46 and (B) the noninsulin‐to‐insulin‐stimulated percent change for phospho S6K1 T389 by Western blot analysis. Blots are normalized to GAPDH for each respective group. *P < 0.05 versus REDD1 WT group, ^†^ *P* < 0.05 versus REDD1 WT Ex group, ^‡^ *P* < 0.05 versus REDD1 KO group. Representative blots are shown. *N* = 5–6 per group.

**Figure 5:**
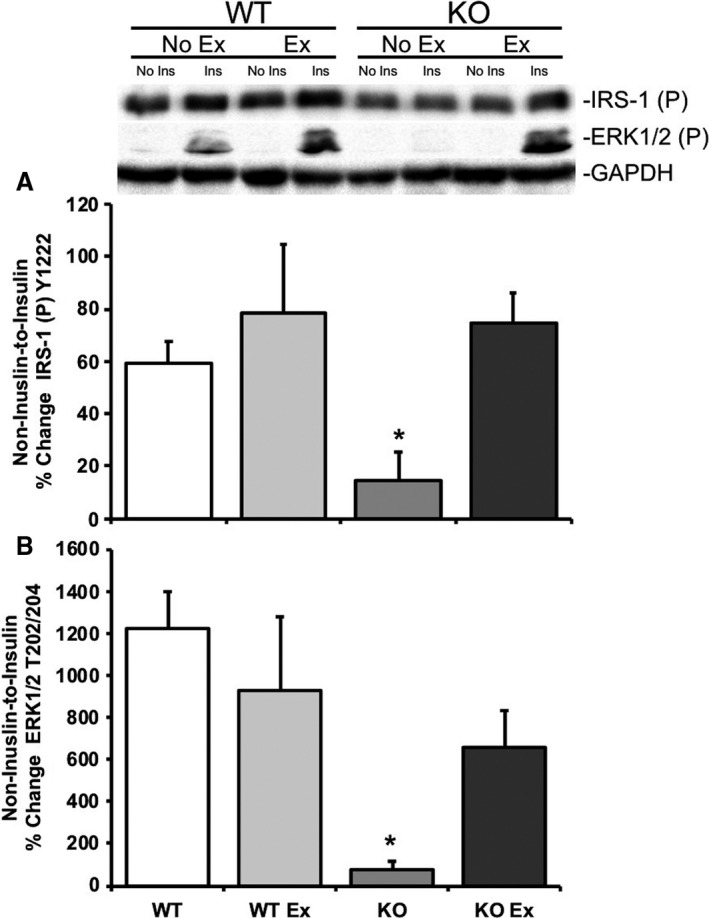
Acute treadmill exercise improves the noninsulin‐to‐insulin‐stimulated IRS‐1 and ERK1/2 phosphorylation in REDD1 KO mouse skeletal muscle. Skeletal muscle from REDD1 WT mice after no exercise −/+acute insulin injection (WT), after an acute exercise bout −/+acute insulin injection in REDD1 WT mice (WT Ex), REDD1 KO mice after no exercise −/+acute insulin injection (KO), and REDD1 KO mice after acute treadmill exercise −/+acute insulin injection (KO Ex) were examined for (A) the noninsulin‐to‐insulin‐stimulated percent change for phospho IRS‐1 Y1222 and (B) the noninsulin‐to‐insulin‐stimulated percent change for phospho ERK1/2 T202/204 by Western blot analysis. Blots are normalized to GAPDH for each respective group. *P < 0.05 versus REDD1 WT group. Representative blots are shown. *N* = 5–6 per group.

Consistent with the rationale that acute treadmill exercise inhibits mTORC1 signaling and improves insulin‐stimulated signaling activation, nonexercised REDD1 KO mice exhibited lower within animal noninsulin‐to‐insulin‐stimulated percent change in skeletal muscle 4E‐BP1 (*P *=* *0.06) and S6K1 (*P* < 0.05) phosphorylation when compared to nonexercised REDD1 WT mice (Fig. 4A and B, respectively). Immediately following an acute bout of treadmill exercise, REDD1 KO exhibited significantly higher (*P* < 0.05) within animal noninsulin‐to‐insulin‐stimulated percent change in 4E‐BP1 and S6K1 phosphorylation when compared to nonexercised REDD1 KO mice (Fig. 4A and B, respectively). Similarly, activation of key insulin signaling kinases in nonexercised REDD1 KO mice exhibit significantly lower (*P* < 0.05) within animal noninsulin‐to‐insulin‐stimulated percent change in IRS‐1 and ERK1/2 phosphorylation when compared to nonexercised REDD1 WT mice (Fig. 5A and B, respectively). Following an acute bout of treadmill exercise, REDD1 KO exhibited a significantly higher (*P* < 0.05) within animal noninsulin‐to‐insulin‐stimulated percent change in IRS‐1 and ERK1/2 phosphorylation when compared to nonexercised REDD1 KO mice (Fig. 5A and B, respectively).

**Figure 6:**
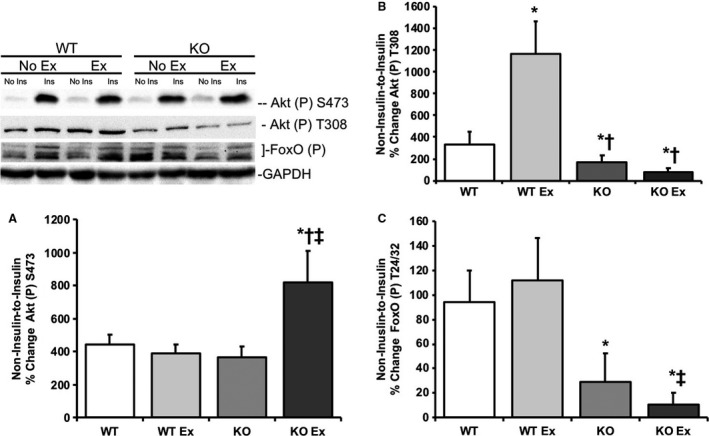
Acute treadmill exercise does not improve the noninsulin‐to‐insulin‐stimulated Akt‐FOXO signaling and key insulin signaling kinase responses in REDD1 KO mouse skeletal muscle. Skeletal muscle from REDD1 WT mice after no exercise −/+acute insulin injection (WT), after an acute exercise bout −/+acute insulin injection in REDD1 WT mice (WT Ex), REDD1 KO mice after no exercise −/+acute insulin injection (KO), and REDD1 KO mice after acute treadmill exercise −/+acute insulin injection (KO Ex) were examined for (A) the noninsulin‐to‐insulin‐stimulated percent change for phospho Akt S473 in WT, KO, and KO Ex mice, (B) the noninsulin‐to‐insulin‐stimulated percent change for phospho Akt T308 in WT, KO, and KO Ex mice, and (C) the noninsulin‐to‐insulin‐stimulated percent change for phospho FOXO1/3A T24/32 in WT, KO, and KO Ex mice. Prior to the calculation of percent change, blots were normalized to GAPDH for each respective group. *P < 0.05 versus REDD1 WT group, ^†^ *P* < 0.05 versus REDD1 WT Ex group, ^‡^ *P* < 0.05 versus REDD1 KO group. Representative blots are shown. *N* = 5–6 per group.

Examining downstream signaling kinases, nonexercised REDD1 KO mice exhibited significantly lower (*P* < 0.05) within animal noninsulin‐to‐insulin‐stimulated percent change in skeletal muscle T308 phosphorylation on Akt and T24/32 phosphorylation on FOXO1/3A when compared to nonexercised REDD1 WT mice (Fig. 6B and C, respectively). Immediately following an acute bout of treadmill exercise, REDD1 KO mice exhibited significantly higher (*P* < 0.05) within animal noninsulin‐to‐insulin‐stimulated percent change in S473 phosphorylation of Akt versus REDD1 WT mice (Fig. 6A). However, there was no change (*P *>* *0.05) in the REDD1 KO within animal noninsulin‐to‐insulin‐stimulated percent change in Akt T308 or FOXO1/3A T24/32 phosphorylation observed following an acute bout of exercise when compared to the respective genotype, nonexercised mice (Fig. 6B and C, respectively). This lack of change in FOXO phosphorylation following insulin stimulation may be a function of significantly elevated (*P* < 0.05) basal phosphorylation of FOXO in the noninsulin, nonexercised REDD1 KO mice when compared to the noninsulin, nonexercised REDD1 WT.

## Discussion

The data presented herein build upon our previous findings that REDD1 is required for normal fasted/fed (Williamson et al. 2014) and insulin‐stimulated growth signaling responses (Dungan et al. 2014). Based upon the reports of Um et al. (2004) and Tremblay et al. (2005), we posited that reduced insulin‐stimulated signaling responses in the absence of REDD1 was due, in part, to negative feedback on IRS‐1 via hyperactive mTORC1. Accordingly, we have reported that prophylactic treatment of REDD1 KO mice with rapamycin prepares skeletal muscle to appropriately respond to acute insulin treatment (Dungan and Williamson 2017). In line with this rationale, acute aerobic exercise effectively inhibits mTORC1 activation (Reynolds et al. 2004) and, subsequently improves the response to insulin (Cartee et al. 1994; Kennedy et al. 1999). Therefore, we sought to determine if an acute bout of treadmill exercise would mitigate the negative effects that a loss of REDD1 has on insulin stimulation. First, this work supports our previous reports showing that REDD1 is required for normal insulin‐stimulated growth signaling. Second, this work provides further insight into the relationship between hyperactive, aberrant mTORC1‐associated signaling and REDD1, in that acute treadmill exercise can partially, but not completely improve the growth signaling response to insulin.

First, the current findings affirm previous reports (Murakami et al. 2011; Hayasaka et al. 2014; Gordon et al. 2017) that a bout of acute treadmill exercise (aerobic exercise) promotes REDD1 expression in REDD1 WT mouse muscle, independent of insulin treatment. While Frost et al. (2009) have demonstrated that insulin promotes skeletal muscle REDD1 expression, this study differed from this work in both dosage and timing of insulin treatment. The upregulation of REDD1 expression during and after treadmill or aerobic type exercise may serve to limit growth signaling (Murakami et al. 2011; Hayasaka et al. 2014) and/or promote transcription of specific genes (Gordon et al. 2017) during times of energetic stress. Similarly, energetic stressors, such as treadmill exercise, promote AMPK activity (Ruderman et al. 2003), which was observed in both the REDD1 WT and KO mice following an acute bout of exercise. Similarly, AICAR treatment is sufficient to inhibit mTORC1 activation through AMPK (Bolster et al. 2002). The current data, along with those of Britto et al. (2014) and Gordon et al. (2017), would suggest that AMPK is functioning properly in REDD1 KO mouse muscle. Accordingly, follow‐up studies may pursue the interplay of AMPK and REDD1 on insulin‐stimulated mTORC1 signaling.

A loss of REDD1 protein expression promotes a significant increase in basal mTORC1 activation (i.e. 4E‐BP1), which have been reported in both mouse embryonic fibroblast (MEF) (Brugarolas et al. 2004) and mice that lack REDD1 (Dungan et al. 2014; Williamson et al. 2014). These findings corroborate our previous findings (Dungan et al. 2014), and that of Regazzetti et al. (2012), in that REDD1 is required for normal insulin‐stimulated signaling. When mTORC1 signaling activation is dysregulated, during basal/fasted conditions, there is improper upstream signaling. These data are also in line with the negative feedback role that S6K1 has on IRS‐1 serine phosphorylation and subsequent degradation (Haruta et al. 2000). The hyperactivation of mTORC1 stabilizes Grb10, subsequently inhibiting insulin‐stimulated signaling through PI3‐kinase/IRS‐1 and ERK1/2 (Yu et al. 2011), which would be consistent with our previous and current findings that REDD1 KO mice have limited IRS‐1 activation following an acute insulin treatment (Dungan et al. 2014). In accordance with these data and our previous report (Dungan and Williamson 2017), a prophylactic treatment with rapamycin reduces the basal hyperactive nature of mTORC1 signaling in REDD1 KO mice. Speaking of the aforementioned AMPK and its inhibitory function on mTORC1 signaling, the expectation during these studies was that mTORC1 signaling would be repressed following the bout of treadmill exercise (Williamson et al. 2006). Accordingly, we observed a significant reduction in mTORC1 activation (S6K1 and rpS6) in WT and KO mice following exercise, with a trending reduction in 4E‐BP1. In REDD1 WT mice, the reduction in mTORC1 activity immediately following exercise was mediated by an increase in both REDD1 expression and AMPK activation, while REDD1 KO mice only had an increased AMPK activation.

Building upon the current and previous findings that AMPK activation functions properly during exercise and treadmill exercise represses mTORC1 in REDD1 KO mouse muscle, we next sought to determine if acute treadmill exercise would restore insulin‐stimulated insulin signaling in REDD1 KO mice. Correspondingly, the (within animal) noninsulin‐to‐insulin‐stimulated percent change in mTORC1 signaling, and IRS‐1 and ERK1/2 phosphorylation of the exercised REDD1 KO mouse muscle was significantly higher when compared to nonexercise REDD1 KO mice and similar to REDD1 WT mice. This finding was similar to our prior work showing that acute rapamycin treatment improved insulin‐stimulated growth signaling activation in REDD1 KO mice (Dungan and Williamson 2017). Here, like the aforementioned rapamycin study, an acute treadmill exercise bout served to reduce basal mTORC1 hyperactivation and derepress mTORC1 inhibitory signals to IRS‐1, preparing the skeletal muscle to appropriately respond to insulin. These data support the premise that a treadmill exercise‐induced increase in AMPK phosphorylation contributed to the reduction in mTORC1 that corresponded with a dramatic increase in IRS‐1 activation following the insulin injection.

However, the current findings show that acute treadmill exercise does not completely reverse all of the negative effects of REDD1 loss on insulin‐stimulated signaling, specifically the Akt‐FOXO axis. A lack of REDD1 hyperactivates mTORC1 signaling, which in turn inhibits IRS‐1 tyrosine phosphorylation and subsequently Akt. The possible difference between the responses of the S473 site and the T308 site with exercise in the REDD1 KO mice may result from mTORC2‐sensitive (rapamycin‐insensitive) and PDK1‐sensitive mechanisms, respectively. mTORC2‐sensitive signaling is not acutely impacted by exercise, unlike mTORC1‐ and PDK1‐sensitive signaling. There are equivocal reports on which Akt sites are more effected by REDD1. Dennis et al. (2014) show that a loss of REDD1 limits insulin's ability to stimulate Akt on the S473 site, whereas phosphorylation of the T308 site was amplified in the REDD1−/− MEFs in response to insulin treatment. This work (Dennis et al. 2014) also reported that REDD1 directly inhibited Akt phosphorylation and activation via increased association of the phosphatase, PP2A, with Akt. However, our previous work (Dungan et al. 2014) and the work from Regazzetti et al. (2012) show that a loss of REDD1 reduces the phosphorylation of Akt on the T308 and the S473 sites. These reports, and that of this study, highlight the complex manner in which REDD1 regulates Akt.

Akt phosphorylation of FOXO represses nuclear localization of FOXO and subsequent promotion of apoptotic and proteolytic target genes (Arden 2004; Stitt et al. 2004; Skurk et al. 2005). Reports are equivocal on the impact that exercise models of this nature (i.e. treadmill running) has on FOXO phosphorylation alone or following insulin stimulation or a fed state (Gwag et al. 2009; Harber et al. 2010; Jamart et al. 2012; Kavazis et al. 2014). Also, the hyperactive basal FOXO1/3A phosphorylation in REDD1 KO skeletal muscle reported herein were consistent with the findings of the Dennis Laboratory (Miller et al. 2018), showing that FOXO1 phosphorylation is elevated in REDD1 CRISPR R28 knockout retinal cells versus WT retinal cells. Given the lack of change in FOXO phosphorylation following insulin treatment in the REDD1 KO when compared to the WT group, it may suggest that there is limited capacity to further enhance FOXO activation. While not examined in these studies, it has been suggested that the flux of FOXO from cytoplasm to nuclei is determined by the cytoplasmic concentration of dephosphorylated FOXO (Schachter et al. 2012). Additionally, it is proposed that the one‐way flux is independent of the nuclear concentration since more FOXO resides in the cytosolic fraction and the rate of efflux of FOXO out of the cytoplasm (Schachter et al. 2012).

These data extend upon our previous findings that a lack of REDD1 reduces skeletal muscle insulin‐stimulated signaling, by showing that acute treadmill exercise is partially sufficient to improve skeletal muscle insulin signaling in mice lacking REDD1. Unlike the REDD1 WT mice, REDD1 KO mice were unable to demonstrate increases in REDD1 with acute exercise, though both WT and KO mice were able to promote an increase phosphorylation of the mTORC1 inhibitory kinase, AMPK. Accordingly, both the REDD1 WT and KO exhibited an inhibition of mTORC1 signaling after the treadmill exercise bout. Acute exercise improved the insulin‐stimulated activation of mTORC1, IRS‐1, Akt on S473, and ERK1/2 in REDD1 KO mice, when compared to nonexercised REDD1 KO mice. However, Akt T308 and FOXO phosphorylation was not altered by acute exercise in mouse muscle that lack REDD1. These findings suggest that a loss of REDD1 promotes discriminate insulin‐stimulated signaling activation of Akt, which could impact FOXO‐mediated transcription that has yet to be determined.

## Conflict of Interest

The authors have no conflict of interest to report.
