# Corrigendum

**DOI:** 10.14814/phy2.14104

**Published:** 2019-06-06

Acute treadmill exercise discriminately improves the skeletal muscle insulin‐stimulated growth signaling responses in mice lacking REDD1

Cory M. Dungan, Bradley S. Gordon, David L. Williamson

Physiol Rep, 7 (4), 2019, e14011, https://doi.org/10.14814/phy2.14011

In the following sentence, the Britto et al. (2014) reference was incorrectly cited:

The current data, along with those of Britto et al. (2014) and Gordon et al. (2017), would suggest that AMPK is functioning properly in REDD1 KO mouse muscle. Accordingly, follow‐up studies may pursue the interplay of AMPK and REDD1 on insulin‐stimulated mTORC1 signaling.

The correct reference used should instead be Britto et al. (2018) both in the body and the reference section.

## References

[phy214104-bib-0001] Dungan, C. M. , B. S. Gordon , and D. L. Williamson . 2019 Acute treadmill exercise discriminately improves the skeletal muscle insulin‐stimulated growth signaling responses in mice lacking REDD1. Physiol. Rep. 7:e14011. 30806987 10.14814/phy2.14011 PMC6383112

[phy214104-bib-0002] Britto, F. A. , F. Cortade , Y. Belloum , M. Blaquière , Y. S. Gallot , A. Docquier , et al. 2018 Glucocorticoid‐dependent REDD1 expression reduces muscle metabolism to enable adaptation under energetic stress. BMC Biol. 16:65. 29895328 10.1186/s12915-018-0525-4 PMC5998563

